# Association between cancer prevalence and use of thiazolidinediones: results from the Vermont Diabetes Information System

**DOI:** 10.1186/1741-7015-5-17

**Published:** 2007-06-21

**Authors:** Maria E Ramos-Nino, Charles D MacLean, Benjamin Littenberg

**Affiliations:** 1 University of Vermont, Department of Pathology, Vermont 05405, USA; 2 University of Vermont, Department of Medicine, Vermont 05401, USA; 3 University of Vermont, College of Nursing and Health Sciences, Burlington, Vermont 05401, USA

## Abstract

**Background:**

Peroxisome proliferator-activated receptors (PPARs) have emerged as important drug targets for diabetes. Drugs that activate PPARγ, such as the thiazolidinediones (TZDs), are widely used for treatment of Type 2 diabetes mellitus. PPARγ signaling could also play an anti-neoplastic role in several *in vitro *models, although conflicting results are reported from *in vivo *models. The effects of TZDs on cancer risk in humans needs to be resolved as these drugs are prescribed for long periods of time in patients with diabetes.

**Methods:**

A total of 1003 subjects in community practice settings were interviewed at home at the time of enrolment into the Vermont Diabetes Information System, a clinical decision support program. Patients self-reported their personal and clinical characteristics, including any history of malignancy. Laboratory data were obtained directly from the clinical laboratory and current medications were obtained by direct observation of medication containers. We performed a cross-sectional analysis of the interviewed subjects to assess a possible association between cancer diagnosis and the use of TZDs.

**Results:**

In a multivariate logistic regression model, a diagnosis of cancer was significantly associated with TZD use, even after correcting for potential confounders including other oral anti-diabetic agents (sulfonylureas and biguanides), age, glycosylated hemoglobin A1C, body mass index, cigarette smoking, high comorbidity, and number of prescription medications (odds ratio = 1.59, *P *= 0.04). This association was particularly strong among patients using rosiglitazone (OR = 1.89, *P *= 0.02), and among women (OR = 2.07, *P *= 0.01).

**Conclusion:**

These data suggest an association between TZD use and cancer in patients with diabetes. Further studies are required to determine if this association is causal.

## Background

Factors affecting cancer incidence in the diabetic population are diverse and complex. Diabetes is a risk factor for colon, pancreas, breast, endometrium and liver cancer in women. In men, although diabetes it is a risk factor for bladder cancer [1,2], it is a protective factor for prostate cancer [3], particularly in those receiving anti-diabetic treatment [4,5]. One of the limitations of such observational studies is that most have not examined the potential association between medication use and cancer risk.

The increasing prevalence of diabetes has intensified the search for new therapies. Orally administered anti-diabetic agents (oral agents) can be used alone or in combination with other oral agents or insulin [6]. Over the last decade, the gamma subtype of the peroxisome proliferator-activated receptors (PPARγ), has emerged as an important drug target in diabetes [7]. PPARγ agonists currently used as oral agents are thiazolidinedione (TZD) drugs, including rosiglitazone and pioglitazone.

The TZDs are attractive options in the therapy of diabetes because they decrease insulin resistance [8-10]. In addition to being a therapeutic option in diabetes, PPARγ agonists are now believed to be a potential strategy for treatment of several cancers [11,12]. Although differential expression of PPARγ is observed in tumors compared to normal tissues, and PPARγ agonists have been shown to inhibit tumor cell growth and survival, the interdependence of these observations is unclear. Conflicting results on the pro-carcinogenic and anti-tumorigenic role of PPARγ agonists such as the TZDs can be found in the literature. Some of the anti-tumorigenic effects of TZDs have been demonstrated in colon cancer cells [13], glioma cells *in vivo *[14], lung cancer cells [15,16], thyroid carcinoma cells [17], hepatocellular carcinoma [18] and others. However, some studies have shown pro-carcinogenic roles for TZDs. Saez et al showed that the use of a TZD increased the number of polyps in the colon of mice [19]. Seargent, Yates and Gill [20] demonstrated that the PPARγ antagonist GW9662 inhibited growth of human mammary tumor cell lines. Surprisingly, GW9662 prevented rosiglitazone-mediated PPARγ activation, but enhanced rosiglitazone-induced growth inhibition [20]. As such, these data support the existence of PPARγ-independent pathways and challenge the belief that TZDs mediate their effects only via activation of PPARγ. These conflicting results must be resolved, as the possible pro-carcinogenic effects of TZDs raise questions about their long-term safety in patients.

Evidence of both pro-carcinogenic and anti-tumorigenic effects of PPARγ agonists have been mainly studied *in vitro *and *in vivo *models that do not take into full account the metabolic characteristics of patients with diabetes. Recently, a population-based report showed that TZDs were associated with reduced risk of lung cancer in diabetic patients [21] in agreement with previous *in vitro *studies [15,16]. Here, we report the association between anti-diabetic therapies, particularly TZDs, and cancer prevalence in diabetic patients participating in the Vermont Diabetes Information System (VDIS).

## Methods

This study is part of a larger project, the Vermont Diabetes Information System (VDIS), a study of 8855 adults with diabetes in primary care practices [22]. The subjects comprised all diabetic adults in 69 practices in Vermont and adjacent New Hampshire and New York. A field survey was completed at study baseline with a sub-sample of subjects in order to provide a better understanding of the non-laboratory features of diabetes. Patients' names were randomly sorted and patients contacted by telephone until a sample of approximately 15% of patients from each practice agreed to participate in the field survey to give a sample of 1007 at the time of analysis. Four patients were dropped from the analysis due to incomplete information, leaving a final sample of 1003.

Subjects completed a questionnaire at home and then were visited by a trained research assistant who reviewed the questionnaire responses, assisted the subject with any missing or unacceptable responses, reviewed the subject's medications, and measured their height and weight using a portable stadiometer and scale. Race, education, income, marital status, functional status, smoking, alcohol consumption and comorbid conditions were obtained by questionnaire. Prior to the interview, patients were instructed to gather all current medications, including over the counter preparations, for review by the research assistant. The medication list was ascertained by direct observation of the medication container with recording of the drug name, dose, frequency and route of administration. Duration of therapy was not recorded.

We used the SF-12 health survey to determine physical and mental functional status. Results were scored and normalized using designed algorithms and expressed in terms of the Physical Component Summary (PCS) and the Mental Component Summary (MCS) [23]. To determine comorbidity, we used a modification of the Self-Administered Comorbidity Questionnaire [24] in which we asked each patient to indicate whether they have had the following conditions: heart attack, heart failure, peripheral arterial disease, stroke, dementia, chronic obstructive lung disease, rheumatic disease, peptic ulcer, cirrhosis, paralysis, renal insufficiency, diabetic vascular complications, AIDS/HIV and depression. All patients also had diabetes, which was not included in the comorbidity count. Patients with two or more comorbid conditions were considered "high comorbidity". Patients were classified as having cancer if they reported any cancer, leukemia, or lymphoma. Specific cancer sites and dates of diagnosis were not recorded.

The interviews occurred between July 2003 and March 2005. Most laboratory data were obtained from the patients' local clinical laboratories, which all use the same Diabetes Control and Complications Trial/Epidemiology of Diabetes Interventions and Complications high performance, liquid chromatography (HPLC) method for the determination of glycosylated hemoglobin (A1C). Less than 1% of A1C tests were performed using the Bayer DCA 2000 immunoassay point of care instrument, which has been shown to compare favorably with the HPLC method [25].

The research protocol was carried out in compliance with the Helsinki Declaration and was approved by the Committee on Human Research of the University of Vermont. The interviewed subjects provided written informed consent. The full study protocol and variables and the medication profiles of the subjects have been previously reported [22,26].

### Statistical approach

We performed a cross-sectional analysis of the interviewed subjects at the time of their enrollment in the VDIS trial. We explored the association between cancer and the use of oral agents, using logistic regression with history of cancer as the outcome variable and the use of TZDs as the primary predictor variable. We then adjusted for possible confounding by social and clinical factors. Potential confounders tested were: other oral anti-diabetic agents (sulfonylureas and biguanides; yes/no), gender, age (years), race (white/other), glycosylated hemoglobin level (A1C; mg %), insulin use (yes/no), body mass index (BMI; kg/m^2^), alcohol drinking (yes/no), cigarette smoking (yes/no), income (in eight ordered categories), duration of diabetes in years, high comorbidity (two or more comorbid conditions), number of prescription medications, number of anti-diabetic drugs, physical function status and mental function status. To reduce the number of variables in the final model, we excluded potential confounders that were associated with the outcome in univariate analyses with *P *> 0.2. Such weak association argues that the variable is unlikely to be a confounder. However, variables representing use of TZDs, sulfonylureas and biguanides were included regardless of their *P*-values. Because diabetes is associated with an increased incidence of breast and endometrial cancers and a reduced incidence of prostate cancers [2-4], we also performed separate multivariate analyses for men and women. Because the effects of TZDs on cancer might be mediated through pathways other than PPARγ, and therefore not be an effect of all TZDs, we also performed separate analyses for pioglitazone and rosiglitazone. We used Stata/SE v.9.2 (StataCorp, College Station, TX, USA) for all analyses.

## Results

The general characteristics of the study population are described in Table 1. The study population is representative of adults with diabetes in primary care practices in Northern New England, USA. Because many of the subjects are over retirement age or suffer disability from chronic conditions, their income is lower than healthy younger Americans.

**Table 1:** Baseline characteristics of 1003 adults with diabetes

Characteristic	n (%) or mean (sd)
History of cancer	126 (12.6%)
TZD therapy	263 (26.2%)
Pioglitazone	136 (51.3%)
Rosiglitazone	128 (48.7%)
Any TZD alone	-71 (27.0%)
TZD and biguanide	-65 (24.7%)
TZD and sulfonylurea	-56 (21.3%)
TZD and biguanide and sulfonylurea	-71 (27.0%)
Gender:	
Men	457 (45.6%)
Women	546 (54.4%)
Age, years	64.8 (12.0)
White ethnicity	973 (97.3%)
Glycosolated hemoglobin A1C, %	7.13 (1.3)
Insulin use	185 (18.4%)
Body mass index, kg/m^2^	33.81 (7.4)
Alcohol use	274 (27.4%)
Cigarette smoking	170 (16.9%)
Median annual income, $	15000–29999
Duration of diabetes, years	10.2 (10.3)
High comorbidity	495 (49.4%)
Number of prescription medications	6.7 (3.8)
Number of anti-diabetic medications	1.2 (0.9)
Physical functional status	41.2 (12.4)
Mental functional status	50.0 (10.7)

TZD, thiazolidinedione; sd, standard deviation; n, number of subjects with the characteristic.

The number of cancer patients in this population was 126 (12.6%). A total of 26% of the subjects used a TZD, including 13.6% on pioglitazone and 12.8% on rosiglitazone (Table 1).

Table 2 presents univariate associations between cancer diagnosis and the other study variables. Rosiglitazone, age, high comorbidity, and number of prescription medications were significantly associated with cancer prevalence.

**Table 2:** Univariate associations between cancer and other patient characteristics

Characteristic	Cancer patients		Non-cancer patients		Odds ratio	*P*
	% or mean (sd)	n	% or mean (sd)	n		
Number of subjects		126		877		
TZD therapy	32.5%	126	25.3%	877	1.42	0.09
Pioglitazone	13.5%	126	13.6%	877	0.99	0.98
Rosiglitazone	19.1%	126	11.9%	877	1.75	0.03
Sulfonylurea therapy	34.9%	126	38.2%	877	0.87	0.48
Biguanide therapy	39.7%	126	40.0%	877	0.99	0.94
Nateglinide therapy	0.8%	126	0.5%	877	1.75	0.62
Men	42.1%	126	46.1%	877	0.85	0.40
Age, years	69.1 (10.2)	126	64.2 (12.1)	877	1.04	<0.001
White ethnicity	97.6%	125	97.3%	875	1.15	0.83
A1C, mean %	7.0 (1.3)	126	7.2 (1.3)	871	0.88	0.12
Insulin therapy	15.9%	126	18.8%	877	0.81	0.43
Body mass index, kg/m^2^	32.7 (6.8)	125	34.0 (7.5)	865	0.97	0.06
Alcohol drinking	25.4%	126	27.6%	876	0.89	0.60
Cigarette smoking	11.1%	126	17.8%	876	0.58	0.06
Median annual income, $	15000–29999	114	15000–29999	813	1.02	0.75
Duration of diabetes, years	10.2 (9.7)	124	10.2 (10.4)	829	1.00	0.98
High comorbidity	71.4%	126	46.2%	877	2.91	<0.001
Number of prescription medications	7.3 (4.3)	126	6.6 (3.7)	877	1.05	0.05
Number of anti-diabetic drugs	1.2 (1.0)	126	1.2(0.9)	877	1.01	0.92
Physical functional status	40.9 (13.7)	125	42.3 (12.7)	871	0.99	0.27
Mental functional status	50.5 (10.5)	126	49.4 (10.9)	875	1.01	0.30

sd, standard deviation; n, number of patients for which data were available; TZD, thiazolidinedione.

Next, potential confounding variables associated with cancer with *P *< 0.2 and all oral agents regardless of their *P*-value were included in a logistic regression model using cancer as the outcome. These included TZD therapy, sulfonylurea therapy, biguanide therapy, age, glycosylated hemoglobin (A1C), BMI (kg/m^2^), cigarette smoking, high comorbidity conditions, and number of prescription medications. This model showed a significant association between cancer and the use of any TZD (OR = 1.59, 95% CI (1.03–2.44), *P *= 0.04) (Table 3). A significant association was found for rosiglitazone (OR = 1.89, 95% CI (1.11–3.19), *P *= 0.02) (Table 3), but not for pioglitazone (OR = 1.09, 95% CI (0.62–1.94), *P *= 0.76) (Table 3). Other variables significantly associated with cancer history include age and high comorbidity (Table 3).

**Table 3:** Multivariate logistic regression: cancer vs. TZD therapy and potential confounders including combined oral anti-diabetic treatments

	Odds ratio	*P*	95% CI
**Any TZD therapy (N = 983)**	1.59	0.04	1.03–2.44
Age	1.03	0.01	1.01–1.05
Biguanide therapy	1.37	0.14	0.90–2.10
Sulfonylurea therapy	0.70	0.09	0.45–1.05
Glycosolated hemoglobin A1C, %	0.91	0.30	0.76–1.08
Body mass index, kg/m^2^	0.97	0.08	0.94–1.00
Cigarette smoking	0.61	0.13	0.33–1.15
High comorbidity conditions	3.40	<0.001	2.14–5.39
Number of prescription medications	1.01	0.97	0.95–1.07

**Rosiglitazone therapy (N = 983)**	1.89	0.02	1.11–3.19
Age	1.03	0.05	1.01–1.05
Biguanide therapy	1.34	0.16	0.89–2.04
Sulfonylurea therapy	0.70	0.10	0.46–1.07
Glycosolated hemoglobin A1C, %	0.92	0.34	0.77–1.09
Body mass index, kg/m^2^	0.97	0.09	0.94–1.00
Cigarette smoking	0.61	0.13	0.33–1.15
High comorbidity conditions	3.32	<0.001	2.10–5.26
Number of prescription medications	1.00	0.92	0.95–1.06

**Pioglitazone therapy (N = 983)**	1.09	0.76	0.62–1.94
Age	1.03	0.01	1.01–1.05
Biguanide	1.41	0.11	0.93–2.13
Sulfonylureas	0.71	0.12	0.47–1.09
Glycosolated hemoglobin A1C, %	0.91	0.27	0.77–1.08
Body mass index, kg/m^2^	0.98	0.12	0.91–1.01
Cigarette smoking	0.62	0.13	0.33–1.15
High comorbidity conditions	3.36	<0.001	2.12–5.32
Number of prescription medications	1.01	0.81	0.95–1.07

TZD, thiazolidinedione.

When the analysis was stratified by gender, it showed a highly significant association between cancer prevalence and TZDs in women (OR = 2.07, 95% CI (1.18–3.63), *P *= 0.01) (Table 4), but not in men (OR = 1.20, 95% CI (0.58–2.46), *P *= 0.63) (Table 4).

**Table 4:** Multivariate logistic regression of dependent variable cancer versus any TZD therapy (Rosiglitazone or Pioglitazone) and potential confounders stratified by gender

	Odds ratio	*P*	95% CI
**Women (N = 535)**			
TZD therapy	2.07	0.01	1.18–3.63
Age	1.02	0.25	0.99–1.04
Biguanide use	1.63	0.09	0.93–2.84
Sulfonylurea use	0.49	0.02	0.27–0.89
Glycosolated hemoglobin A1C, %	0.94	0.59	0.76–1.17
Body mass index, kg/m^2^	0.97	0.07	0.93–1.00
Cigarette smoking	0.43	0.06	0.18–1.03
High comorbidity conditions	2.90	0.001	1.56–5.41
Number of prescription medications	1.09	0.03	1.01–1.18

**Men (N = 448)**			
TZD therapy	1.20	0.63	0.58–2.46
Age	1.05	0.01	1.01–1.08
Biguanide use	0.99	0.97	0.51–1.91
Sulfonylurea use	1.22	0.56	0.63–2.35
Glycosolated hemoglobin A1C, %	0.83	0.22	0.62–1.12
Body mass index, kg/m^2^	0.97	0.32	0.91–1.03
Cigarette smoking	0.78	0.62	0.30–2.03
High comorbidity conditions	4.29	<0.001	2.11–8.72
Number of prescription medications	0.89	0.018	0.80–0.98

TZD, thiazolidinedione.

TZDs are used alone or in combination with other oral anti-diabetic agents (Table 1). The multivariate analyses found no evidence for the use of other oral agents confounding the relationship of TZD therapy with cancer (Table 3), but in the gender stratified analysis, the use of sulfonylurea is negatively associated with cancer in women (OR = 0.49, 95% CI (0.27–0.89),*P *= 0.02) (Table 4). Other variables significantly associated with cancer history in women included high comorbidity (OR = 2.90, 95% CI (1.56–5.41),*P *= 0.001) and number of prescription medications(OR = 1.09, 95% CI (1.01–1.18),*P *= 0.03) (Table 4). Similarly, in men, high comorbidity (OR = 4.29, 95% CI (2.11–8.72),*P *< 0.001) and number of prescription medications(OR = 0.89, 95% CI (0.80–0.98),*P *= 0.02) were associated with cancer history (Table 4).

## Discussion

We have demonstrated an association between the use of TZDs and cancer in a community-based population of adults with diabetes. The association was observed primarily among rosiglitazone users and not among subjects using pioglitazone. Furthermore, the association was found in women, but not men.

The gender-dependent observation is difficult to explain. TZDs have gained widespread use in polycystic ovary syndrome (PCOS) [27] that has been correlated with endometrial cancer [28]. Perhaps the TZDs are not causing cancer, but are used by patient with cancers caused by PCOS. It is worth noting that as the average age in this population is post-menopausal, the use of TZDs for PCOS is probably low. Unfortunately, we do not have data on the history of PCOS in these patients. More alarmingly, it is possible that TZDs disrupt estrogen pathways and increase the incidence of hormone-sensitive tumors such as those of the breast and endometrium. We do not know which specific tumors are increased in the TZD users in this population.

Likewise, we are not aware of a convincing explanation or previous results to support the finding in this study of an association with cancer for rosiglitazone, but not pioglitazone. As both are thought to activate PPARγ with similar potency, this finding suggests another mechanism for the association. Furthermore, we cannot explain the differences found between the anti-cancer effect of PPARγ activation *in vitro *and our results. Some studies suggest that natural mutant PPARγ alleles might impair native PPARγ function [29] and differences in the PPARγ genotype could modify the response to TZD treatment. Further studies incorporating rigorous controls for genetic and environmental confounders are required to identify the molecular mechanisms involved in the differences found between the two drugs and the discrepancies with *in vitro *studies.

Cancer patients used slightly more prescription medications than non-cancer patients, but similar numbers of anti-diabetic medications (Table 2). This is consistent with the need for additional anti-cancer therapies as determined from the prescription drug list of cancer patients compared to non-cancer patients. Cigarette smoking is somewhat less prevalent in the cancer patients than the non-cancer patients, in spite of the well-known association of tobacco use and cancer incidence. This could be because cancer survivors are highly motivated to quit smoking [30], or that smokers with cancer die at a higher rate than non-smokers with cancer [31].

We note that cancer patients tended to have more comorbid conditions than non-cancer patients. This could be due to the well-known associations between some of the specific measured comorbidities and various cancers. For instance, chronic obstructive lung disease and cirrhosis are often associated with lung cancer and hepatoma, respectively [32,33].

To further address potential confounding, we also included two other common oral anti-diabetic agents (sulfonylureas and biguanides). Results presented here showed no significant association of either class of medications with cancer prevalence or any confounding of the association between TZDs and cancer in the general model (Table 3). However, a protective effect of sulfonylurea therapy for women was found in the gender-stratified model (Table 4). The fact that we observe an association with cancer for some PPARγ agonists (TZDs), but not others (sulfonylureas; [34]) suggests the notion of PPARγ-independent mechanisms in the association between TZDs and cancer.

This study has several strengths. First, the interviewed subjects were a randomly selected subset of a large population of patients receiving care in the Northeast USA, and are therefore likely to be representative of primary care patients. Second, all data on medications were obtained by direct observation in the patient's home and not from secondary sources.

This study does however also have several limitations, including lack of confirmation of the cancer diagnoses, and lack of information on the time relation between onset of cancer and the use of TZDs or the duration of treatment. In addition, information on the type or stage of the cancer cases is missing, and skin cancers are not reported. As in any cross-sectional study, unmeasured confounders could be responsible for the apparent associations found.

## Conclusion

Our findings suggest a possible association between cancer and the use of TZDs, particularly rosiglitazone, and particularly among women, but this association requires further investigation.

## Competing interests

The author(s) declare that they have no competing interests.

## Authors' contributions

All authors contributed equally to this work

## Pre-publication history

The pre-publication history for this paper can be accessed here:


